# Summer Complaints

**Published:** 1892-10

**Authors:** 


### SUMMER COMPLAINTS.

One of the most common complaints which occur in warm weather is the result of food poisoning. Of course, it is well known that under the influence of heat, very many foods change quickly, and become unfit to eat; but comparatively few, however, are aware that frequently during such changes poisons are generated.

It is unnecessary to consider the nature of these poisons, and how they are produced; it will suffice to say that some of them are of the most virulent character, and more powerful even than strychnine. Of all perishable foods, probably milk is the most dangerous under certain conditions. Unfortunately, also, it is extremely difficult to detect just when these conditions exist.

Milk may sour, and still be innocent of harm. In fact, in this changed state it is frequently used in cooking, and eaten in the form of "Dutch cheese." And yet milk may become to an intense degree poisonous, even before a change appreciable to the ordinary examiner has occurred.

The special influence which render milk poisonous are heat and foul air. Heat alone would scarcely be sufficient, for it would do little more than promote the acid changes or souring; but when combined with the impurities which so often float in the air, its evil effects are greatly intensified.

Moisture is also influential in promoting the generation of poison in milk; and, perhaps it is scarcely less active that heat and foul air. There is a popular conviction that thunder storms cause milk to sour quickly. This is certainly a fact; moreover, in a great degree, they favor the other and poisonous changes.

Persons who are the most likely to suffer from milk poisoning are they who live in unhealthly locations, where there is more or less filth to poison the air with its emanations; or tenants of poorly ventilated rooms, the atmosphere of which must necessarily be impure. Lack of cleanliness, also, either within or about the homes, or in the habits of the inmates, is an urgent invitation to milk poisoning. And these baneful influences are scarcely less influential in causing other foods to undergo decomposition and become poisonous.

Perhaps it would be well to go a little further in this direction, and more minutely into some of the most hurtful conditions. A family living near a stable, and within its unpleasant atmosphere, would need to use especial care in keeping their milk. Or if a house stood alone, and far removed from any other building, and yet the grounds were allowed to become untidy and littered up here and there with kitchen waste, etc., then milk poisoning, might with good reason, be expected by its occupants.

A housekeeper may keep her rooms neat and clean, and yet be in danger of this accident, if she failed through ignorance or neglect to properly care for the cans, pitchers, etc., in which she stored her milk, or to frequently wash out and air her ice chest. In a word, in order to prevent milk from undergoing poisonous changes, it must be kept in clean dishes, in a cool place, and away from foul air.

It is the custom with many housekeepers to allow their milk to remain until used in the cans in which it is received, the same, of course, being put on ice, if there is any in the house. Others pour the milk from the cans into pitchers, and set them away in the ice chest. Both of these methods are open to many objections, and the last is especially unwise, for the milk being in an open vessel soon absorbs the odors from the other foods stored with it.

The only proper way to treat milk is this : Where all the members of the family are adults, provide several pint bottles, round in shape, and of clear white glass, so that they may be easily cleaned, and it will be easy to see when they are perfectly clean. The number of bottles should be large enough to make up two sets, for use on alternate days. And all should be provided with rubber corks, which can be obtained at drug stores.

As soon as possible after the milk is delivered it should be poured into these bottles, which have been previously cleaned "on honor." A kettle should be placed upon the stove, over a fire, and in this a collander. As soon as the water boils, the bottles should be placed, uncorked in the collander, and under a cover.

The milk ought to steam at least an hour. And as the operation is going on and while the breakfast is cooking, or its dishes are being washed, it does not entail any trouble or expense. The steaming destroys all impurities which happen to be in the milk ; and at the same time it renders it less liable to sour or undergo poisonous changes.

When the milk has been steamed long enough, the corks, which have been standing a few moments in boiling water, should be pressed in tightly. Now, all that remains is to set the bottles in the ice chest and surround them with ice. Several small bottles instead of one large one are recommended, for the obvious reason that there will be less exposure of the milk to the air, one bottle being opened and used while the others remain safely in the ice chest.

After the bottles have been emptied, they should be well rinsed in cold water, then carefully washed, again rinsed, and filled with fresh water to which has been added a little baking soda. This should remain in them until the following morning, when they should be freely rinsed and put into the hot oven, there to wait while the milk is steaming.

This method is simple and easily practised. It really demands but a few moments of actual work, and yet affords a security against a serious and sometimes fatal danger that cannot be obviated in any other way.

The poison which is generated in milk, known as tyrotoxicon, finds its readiest victims among young children. Adults have a certain amount of resistant power which the little ones do not possess ; therefore, a child might be poisoned by milk taken from the same vessel from which the parents had drawn and suffered no ill effects.

The symptons of milk poisoning in adults are essentially the same as appear in severe cholera morbus ; while in children they are identical with those of cholera infantum. As a matter of fact, there is ample reason for the belief that these so-called summer complaints, in most instances, are pure and simple attacks of milk poisoning.

Physicians occasionally encounter cases of cholera morbus in men and women but a few hours previous strong, robust and healthy, which run a course as rapidly fatal as that terrible scourge cholera. In these cases there is intense prostration, and the fires of life seem almost extingushed, for the skin is cold and clammy, and no matter what treatment is applied, it cannot be made warm again.

This condition called collapse, is threatened in all severe cases of milk poisoning ; and, not unlikely, in many of the fatal cases of what passes for cholera morbus, the actual cause is an unusually large quantity of this virulent poison. It is, of course, too much to say that all attacks of cholera morbus and cholera infantum are due to this terrible agent, and yet the chances are that but few cases, comparatively, occur from other causes.

In milk poisoning, the symptons that first present themselves are nausea and colicky pains. As a rule, they come on quite suddenly, and usually in the course of from two to four hours after eating. If a person's stomach is weak, he generally soon vomits ; but he may escape this if diarrhœa comes on quickly. This speedily becomes excessive and very weakening, and the discharges are but little more than faintly colored water. In the meantime, the pain keeps up or grows decidedly worse. If the victum was hardy and strong when attacked, and the diarrhœa brisk at first, he usually quickly recovers after the bowels have expelled the poisons; feeble persons, however, are generally several days in regaining their usual health.—*Boston Journal of Health.*

